# Oncolytic Virotherapy as Emerging Immunotherapeutic Modality: Potential of Parvovirus H-1

**DOI:** 10.3389/fonc.2014.00092

**Published:** 2014-05-01

**Authors:** Markus Moehler, Katrin Goepfert, Bernd Heinrich, Caroline J. Breitbach, Maike Delic, Peter Robert Galle, Jean Rommelaere

**Affiliations:** ^1^1st Department of Internal Medicine, University Medical Center of the Johannes Gutenberg, University of Mainz, Mainz, Germany; ^2^Jennerex Biotherapeutics, Inc., San Francisco, CA, USA; ^3^Division of Tumor Virology, German Cancer Research Center (DKFZ), Heidelberg, Germany

**Keywords:** immunotherapy, autonomous parvovirus, H-1PV, talimogene laherparepvec, T-VEC, JX-594, dendritic cells, CTLA-4

## Abstract

Human tumors develop multiple strategies to evade recognition and efficient suppression by the immune system. Therefore, a variety of immunotherapeutic strategies have been developed to reactivate and reorganize the human immune system. The recent development of new antibodies against immune check points may help to overcome the immune silencing induced by human tumors. Some of these antibodies have already been approved for treatment of various solid tumor entities. Interestingly, targeting antibodies may be combined with standard chemotherapy or radiation protocols. Furthermore, recent evidence indicates that intratumoral or intravenous injections of replicative oncolytic viruses such as herpes simplex-, pox-, parvo-, or adenoviruses may also reactivate the human immune system. By generating tumor cell lysates *in situ*, oncolytic viruses overcome cellular tumor resistance mechanisms and induce immunogenic tumor cell death resulting in the recognition of newly released tumor antigens. This is in particular the case of the oncolytic parvovirus H-1 (H-1PV), which is able to kill human tumor cells and stimulate an anti-tumor immune response through increased presentation of tumor-associated antigens, maturation of dendritic cells, and release of pro-inflammatory cytokines. Current research and clinical studies aim to assess the potential of oncolytic virotherapy and its combination with immunotherapeutic agents or conventional treatments to further induce effective antitumoral immune responses.

## Introduction

Human tumors develop complex strategies to circumvent the human immune system and to become resistant to classical therapies like radiotherapy or chemotherapy (1). Besides the low immunogenicity of tumors, tumor-induced dysregulation of the immune response leads to loss of effective immune defense and uncontrolled tumor growth. Even though many classical chemotherapy or radiation strategies induce some extent of tumor surveillance (1), new approaches should be tested to overcome early tumor resistance and recurrence. Thus, the basic challenge of molecular immune targeting is to conquer local regulatory mechanisms in order to re-introduce tumor immune recognition and promote tumor cell apoptosis and immunogenic cell death (ICD) (2). Recently, loss of immune defense has been shown to be caused by expression of different immune suppressive receptors also called immune checkpoint pathways, such as cytotoxic T-lymphocyte antigen-4 (CTLA-4) (3). Its ligation is crucial to preventing immune overreaction by inhibiting T-cell activation (4). The inhibitory CTLA-4 antibody ipilimumab [Yervoy, Bristol Myers Squibb (BMS)], approved for the treatment of metastatic melanoma patients, blocks this negative immune stimulatory receptor, thereby preventing downregulation of T-cell activation (5).

Oncolytic virotherapy represents an emerging therapeutic modality that has achieved tumor regression in several pre-clinical models and in clinical trials (6). Preferential depletion of cancer cells by oncolytic viruses (OV) is based on the fact that more aggressive tumor cells show both impaired antiviral responses and higher permissiveness for virus replication. Therefore, these agents open up new horizons for the treatment of cancer types that commonly display poor prognosis (7, 8). Cancer virotherapy is an old concept that arose from observations of unexpected tumor regressions coinciding with virus infections. This can be exemplified by a report on Newcastle disease virus (NDV) in gastric cancer dating back to 1971 (9). It should be stated that viruses with natural or engineered effects on the immune system are highly potent candidates for cancer therapy (Table 1). Herein, oncolytic viruses can be engineered to deliver therapeutic transgenes to cancer cells, causing additional anti-tumor effects through cytokine secretion and induction of anti-tumor immune responses (10–14). For example, the oncolytic vaccinia virus pexastimogene devacirepvec (Jennerex, Inc., and Transgene SA; Pexa-Vec, JX-594) and herpes simplex virus (HSV) talimogene laherparepvec (T-VEC, Amgen) were “armed” with GM-CSF-expressing genes (15, 16) to initiate local and systemic immune responses. Recently a randomized, Phase III trial of talimogene laherparepvec or GM-CSF in patients (pts) with unresectable melanoma with regional or distant metastases (OPTiM) met its primary endpoint by improving durable response rates versus GM-CSF alone, and showed a tolerable safety profile (17). A Phase II study of Pexa-Vec in primarily first-line liver cancer (HCC) patients demonstrated survival improvement in patients receiving intratumoral (it) injections of high-dose Pexa-Vec (18). The following randomized Phase IIb study in second-line HCC patients did not meet its primary endpoint of survival improvement for Pexa-Vec compared to best supportive care (BSC) (19). However, this trial was comprised primarily of patients with end-stage disease and significant comorbidities such as liver cirrhosis, therefore likely not the optimal population for successful OV therapy. Therefore, further studies of Pexa-Vec in a less advanced HCC population as well as other indications are warranted. Besides above-mentioned agents, various other viruses were shown to have oncolytic and/or immunostimulating properties, and are presently used in clinical trials. These include Parvovirus, Adenovirus, Vesicular Stomatitis Virus, Reovirus, NDV, Measles Virus, Seneca Valley Virus, Poliovirus, and Coxsackie Virus (Table 1).

**Table 1:** **Oncolytic viruses**.

Oncolytic virus	Family	Pre-clinical data	Clinical trial	Selected reference
Parvovirus H-1	Parvoviridae ss DNA Icosahedral capsid	Oncotoxicity of the viral protein NS1 Virus replication-associated cytopathic/lytic effects Activation of immune responses Transgene expression (cyto/chemokines) Inhibition of neo-angiogenesis Ref. (12–14, 20–26)	Phase I/IIa glioblastoma multiforme (ParvOryx01)	Clinical: NCT01301430 (27)
Vaccinia/poxvirus	Poxviridae ds DNA Enveloped Pexastimogene devacirepvec (Pexa-Vec; JX-594): engineered from Wyeth vaccine strain GLV-1h68 (GL-ONC1): engineered from vaccinia virus Lister strain	Cell lysis caused by viral replication Thymidine kinase (TK) gene-inactivated, selective replication Transgene expression (GM-CSF) (28) Disruption of tumor-associated vasculature (29) Induction of antibody-mediated complement-dependent cancer cell lysis (30)	Phase IIB, hepatocellular carcinoma, Pexa-Vec Phase II, colorectal cancer, Pexa-Vec Phase II renal cell carcinoma, Pexa-Vec Phase I and II, malignant pleural effusion, peritoneal carcinomatosis (GL-ONC1)	Clinical: NCT01387555; NCT01394939; NCT01766739; NCT01443260
HSV-1	Herpesviridae ds DNA Icosahedral capsid Enveloped Talimogene laherparepvec: engineered from JS1 strain	Cell lysis caused by viral replication ICP34.5 functional deletion (neurovirulence factor) ICP47 deletion Activation of anti-tumor immunity Transgene expression (GM-CSF) (31)	Phase III complete, malignant melanoma (talimogene laherparepvec)	Clinical: NCT00769704 (32, 33)
Adenovirus	Adenoviridae ds DNA Oncorine based on H101-virus	Cell lysis caused by viral replication Activation of anti-tumor immunity Cytotoxicity by viral proteins (E4ORF4) (34) Transgene expression (GM-CSF by CG0070) (35, 36)	Phase II and III, bladder cancer (CG0070) Approved therapeutic (China), head and neck cancer (Oncorine)	Clinical: NCT01438112 (37, 38)
Vesicular stomatitis virus (VSIV, often VSV)	Rhabdoviridae ss RNA	Expression of IFN-β (39, 40)	Phase I, liver cancer (IFN-β expressing VSV)	Clinical: NCT01628640
Reovirus	Reoviridae ds RNA Icosahedral capsid	Cytopathic effect Activation of immune response (41)	Phase I-III, several entities, e.g., head and neck cancer, non-small cell lung cancer, prostate cancer, colorectal cancer (Reolysin)	Clinical: NCT01166542; NCT01708993; NCT01619813; NCT01622543
Newcastle disease virus	Paramyxoviridae ssRNA	Activation of anti-tumor immunity (42–47)	Phase I and II study in glioblastoma, sarcoma and neuroblastoma	Clinical: NCT01174537
Measles virus	Paramyxoviridae ss RNA	Cytopathic effect (48) Anti-tumor activity (49)	Phase I study in malignant solid tumor, breast cancer, malignant tumor of colon, GIST, ovarian cancer Phase I study in multiple myeloma and plasma cell neoplasm Phase I study in metastatic squamous cell carcinoma of the head and neck cancer Phase I in malignant pleural mesothelioma Phase I in brain and central nervous system tumors Phase I in ovarian cancer, peritoneal cavity cancer Phase I and II study in recurrent ovarian cancer	Clinical: NCT01376505; NCT00450814; NCT01846091; NCT01503177; NCT00390299; NCT02068794 (50–52)
Seneca valley virus	Picornaviridae ss RNA	Antineoplastic activity (53)	Phase I safety study, solid tumors with neuroendocrine features Phase II after chemotherapy in small cell lung cancer Phase II with cyclophosphamide in neuroblastoma, rhabdomyosarcoma	Clinical: NCT00314925; NCT01017601; NCT01048892 (54)
Cavatak virus (Coxsackie virus A21)	Picornaviridae ss RNA Capsid		Phase I study in non-small cell lung cancer, castrate resistant prostate cancer, and melanoma and bladder cancer Phase I study in melanoma, breast, and prostate cancer Phase I study in melanoma Phase I study in head and neck cancer Phase II study, malignant melanoma	Clinical: NCT02043665; NCT00636558; NCT00438009; NCT00832559; NCT01227551; NCT01636882

*Oncolytic viruses in clinical trials (ds, double stranded; ss, single stranded)*.

**Figure 1:**
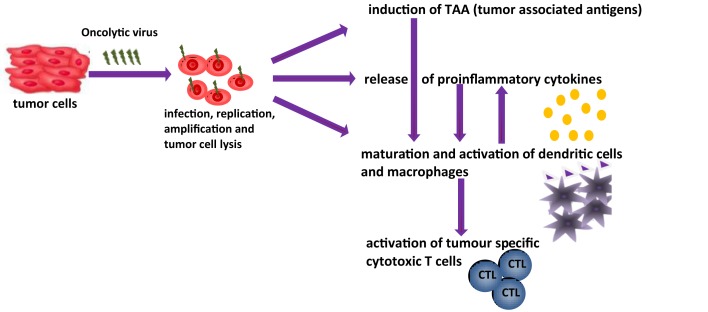
**Oncolytic viruses and their possible function in tumor therapy [changed after Ref. (14)]**.

**Figure 2:**
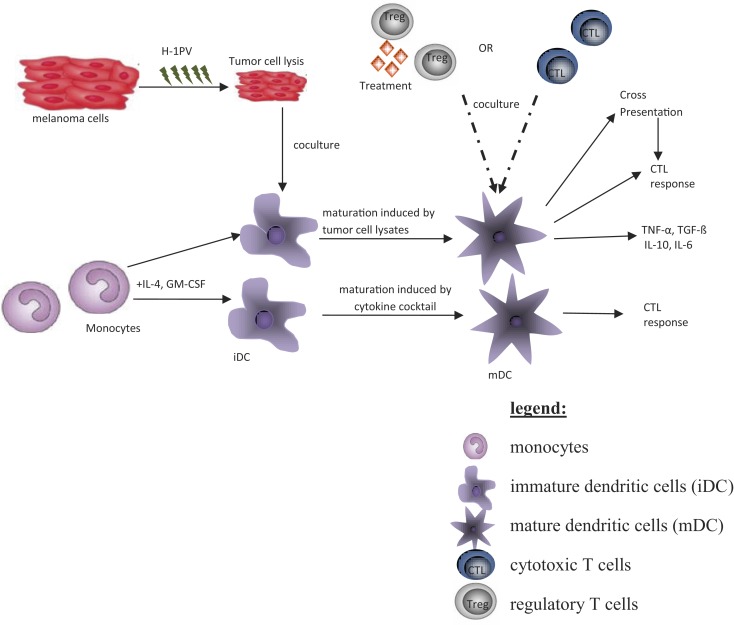
**The *ex vivo* human melanoma model**.

The aim of this article is to provide an overview of upcoming oncolytic viruses and their potential immunogenic therapeutic effects. A first insight into this issue is provided through our pioneer studies showing that infection with the autonomous parvovirus H-1 (H-1PV) generated immunogenic tumor cell lysates (TCLs) (14). H-1PV-infected TCLS proved able to induce maturation of dendritic cells (DCs), release of pro-inflammatory cytokines, tumor-associated antigens (TAA) cross-presentation, and T-cell stimulation in an *ex vivo* human melanoma model (see Figures 1 and 2) (7, 14, 55, 56). On the basis of these observations, we present the prospects of H-1PV and other OVs activating the human immune system either alone or in combination with immunomodulators, such as antibodies blocking immune suppressive receptors.

## Methods

The human *ex vivo* melanoma model (Figure 2) represents a system that mimics the *in vivo* situation (14). Thus, it was used to investigate effects of H-1PV-infected or tremelimumab-treated tumor cells on immune activation. The human melanoma cells MZ7-Mel, SK29-Mel-1, and SK29-Mel-1.22 used were a gift from T. Woelfel (Mainz, Germany) (57). The SK29-Mel-1.22 cell line (A2^−^) is an *in vitro* selected HLA-A2-loss variant of HLA-A2-positive SK29-Mel-1 (A2^+^) line (58, 59). The cytotoxic T-cell clones CTL2/9 and CTL IVSB recognize different antigens of SK29-Mel-1 cells in association with HLA-A2 (57, 58), lyse SK29-Mel cells, and release interferon γ (IFNγ) upon specific recognition of SK29-Mel-specific TAA (58).

Peripheral blood mononuclear cells (PBMCs) were derived from buffy coats of healthy blood donors. Monocytes were isolated via adherence, and differentiation into immature DCs (iDCs) was achieved by stimulation with GM-CSF and interleukin-4. Matured DCs (mDCs) were generated by stimulation with a cytokine cocktail for 2 days (60). For coculture experiments, melanoma cells were kept in FCS-free medium. For induction of maturation and phagocytosis, tumor cells were co-cultured with iDCs at a ratio of 1:3 for 2 days. CTL-Coculture with DC was performed at 1:10 ratio (60).

## Results: Oncolytic Viruses are Able Not Only to Kill Human Tumor Cells but also to Stimulate Anti-Tumor Immune Responses: The Case of Parvovirus H-1PV

Over the last years, OV therapy has shown promising results in both pre-clinical and clinical studies against various solid tumors (61). It is worth noting that besides their own anti-tumor efficiency, OVs can resensitize resistant tumors to chemotherapeutics, thereby highlighting the potential of OVs in multimodal treatments (12, 13). We were particularly interested in the oncolytic parvovirus H-1PV [for reviews, see Ref. (20, 62)]. The mode of action of H-1PV involves both direct oncolytic and immune-mediated components, making this virus an attractive candidate for inclusion in the cancer immunotherapy armamentarium (60). H-1PV is a small nuclear-replicating DNA virus, which preferentially multiplies in oncogene-transformed and tumor-derived cells (7). This oncotropism results at least in part from the dependence of H-1PV on proliferation and differentiation factors that are dysregulated in neoplastic cells (20). In consequence, H-1PV exerts oncolytic effects, which were documented in human cells from various tumor entities including melanoma, pancreatic (PDAC), hepatocellular (HCC), colorectal or gastric carcinomas, sarcoma, glioma, and other neuroectodermal tumors (7, 20, 21, 62–64). Most interestingly, the death mechanisms activated by parvoviruses allow them to overcome resistance of tumor cells to conventional cytotoxic agents (22, 65). Another intriguing aspect of H-1PV-mediated OV lies in the possibility of combining H-1PV with conventional cytotoxic drugs to achieve synergistic tumor cell killing effects, as demonstrated for instance in the PDAC system (13, 21, 22, 66).

Though not or poorly infectious for humans under natural conditions, H-1PV can be administered experimentally to patients, resulting in viremia and seroconversion (67). Infections with H-1PV appear to be clinically silent (68). It should also be stated that recombinant parvoviruses can be constructed, for example to transduce immunostimulatory cytokines (62). This arming strategy was found to increase the anti-tumor effects of parvoviruses in certain models (69–71).

### Bringing H-1PV from the bench to the bedside

Recent work using an immunocompetent rat glioma model showed that H-1PV was able to efficiently cure gliomas, while raising an anti-tumor memory immune response. This oncosuppressive effect appears to rely on both the direct oncolytic activity of H-1PV and its handover to the host immune system (23). These pre-clinical data led to the current clinical evaluation of H-1PV it and intravenous (iv) administration to patients with recurrent resectable GBM progressing in spite of conventional therapies (27).

### H-1PV-induced tumor cell lysates trigger maturation of iDCs and exert immunostimulating effects

H-1PV had little direct killing activity on human immune cells *in vitro*, in particular APCs and CTLs. Interestingly, the analysis of infected PBMCs revealed the induction of markers of both macrophage and Th1cell activation (Table 2). This Th1 bias is indicative of a possible direct immunostimulating capacity of the parvovirus. Nevertheless, a major impact of H-1PV on the immune system appears to be indirect, i.e., mediated by infected tumor cells, as discussed in the following sections. H-1PV caused the death of human melanoma cells in culture, including the above-mentioned SK29-Mel-1 and SK29-Mel-1.22 lines. The extent of cell killing varied between tested lines, was dependent on the multiplicity of infection (MOI) and correlated with expression of the replicative viral non-structural protein NS1. In this system, H-1PV induced an apoptotic cell death, which was accompanied with the release of immunogenic HSP72 (63).

**Table 2:** **Direct immunostimulating effects of parvovirus H-1PV**.

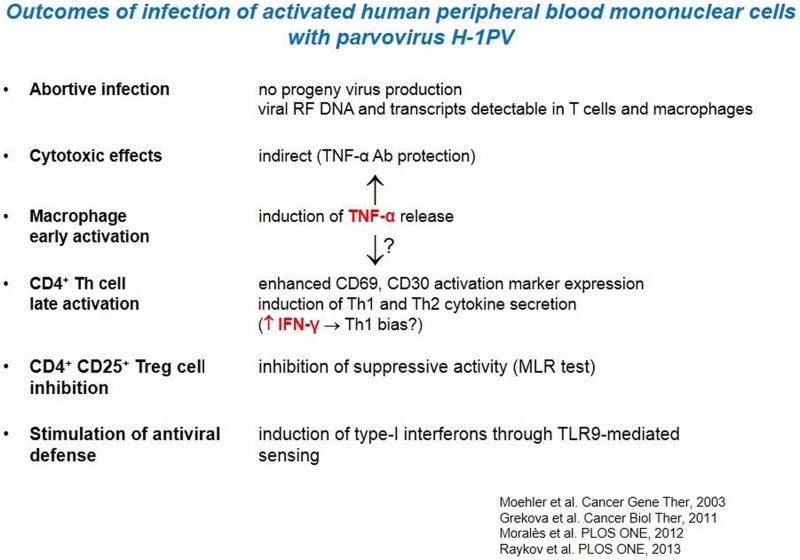

In further experiments it was shown that H-1PV-infected melanoma TCLs were phagocytosed by iDCs and induced their maturation, in particular the secretion of pro-inflammatory cytokines such as TNFα and IL-6 (13, 63). Lysates of infected SK29-Mel-1.22 and MZ7-Mel cells were both competent for inducing DC maturation, although the former were more potent than the latter in this regard (13, 14). Primary immune cells were not permissive for H-1PV infection. Little direct killing effect, no apoptosis, and no progeny virus production could be detected in infected lymphocytes, monocytes, immature, and mature DCs (Table 2) (63).

We also demonstrated that human DCs coincubated with H-1PV-induced melanoma TCLs showed enhanced expression of TLR3, TLR9, and other maturation markers. This suggested that virus-induced TCLs contained molecular patterns triggering TLR signaling in DCs, as further evidenced by increased NF-κB levels and production of pro-inflammatory cytokines (12). Some of these immunostimulating patterns may consist of viral constituents, given the known ability of TLR3 and TLR9 for sensing viral determinants.

Combination of the oncolytic virus with cytostatic (cisplatin, vincristine) or targeted (sunitinib) drugs resulted in a further increase in melanoma cell apoptosis but failed to strengthen maturation of DCs. It was verified that the cytotoxic or targeted drug regimen used did not interfere with H-1PV infection (13). Interestingly, the interleukin profile of DCs was altered upon exposure to H-1PV plus sunitinib-cotreated TCLs. It therefore appears that H-1PV combination with this anti-angiogenic drug may reinforce its capacity not only for jeopardizing tumor cell survival but also for modulating the immune system.

### H-1PV induce activation of antigen-specific cytotoxic T-cells and other anti-tumor immune effectors

To further assess whether phagocytosis of H-1-infected TCLs by DCs induces cross-presentation of TAAs to antigen-specific CTLs in an HLA-class I-restricted manner, the above-mentioned human melanoma *in vitro* model was used (58, 72). Both melanoma-specific CTL clones tested were found to release increased levels of IFNγ after being co-cultured with DCs preincubated with H-1PV-infected SK29-Mel-1 or HLA-negative SK29-Mel-1.22 cells (14). Thus, H-1PV-induced TCLs stimulated cross-presentation of TAAs by DCs. This effect may contribute to reinforce the anti-tumor immune response by generating tumor-specific CTLs (14). In addition, several H-1PV-infected tumor cells were recently found to acquire an enhanced capacity for activating NK cells and getting killed by these cells (73, 74). The adjuvant effect of H-1PV was also evidenced *in vivo* by the virus-enhanced efficacy of an autologous tumor cell vaccine (24) and the adoptive transfer of anti-tumor immune cells from animals undergoing oncolytic H-1PV therapy (75).

### Oncolytic H-1PV virotherapy can be combined with immunotherapeutic agents to enhance treatment efficacy

Recent evidence for the expression of the immunosuppressing molecule CTLA-4 on regulatory T-cells (Tregs) and tumors generated widespread interest in the role of CTLA-4 in tumor escape and peripheral tolerance (3, 58). In particular, the human colon adenocarcinoma line SW480 was found to express CTLA-4 on the cell surface. This prompted us to extend the analysis of H-1PV anti-tumor effects to the SW480 system in combination with the anti-CTLA-4 antibody tremelimumab. When applied alone, this antibody had no detectable effect on SW480 cell viability and DC maturation. On the other hand, H-1PV alone was able to kill SW480 cells in a MOI-dependent manner. H-1PV-induced SW480 TCLs triggered iDC maturation in coculture experiments, as revealed in particular by increased release of the pro-inflammatory cytokines IFNγ, TNFα, and IL-6 (64). The secretion of IFNγ was stimulated to a low extent by treatment of the coculture with tremelimumab, recommend the use of the H-1PV/tremelimumab combination treatment to enhance tumor immunogenicity through both DC activation and CTLA-4 masking. It should also be stated that other (immuno)modulators, namely IFNγ (75) and HDAC inhibitors (76), were recently reported to cooperate with H-1PV for tumor suppression in human carcinoma animal models.

## Clinical Evidence of OV-Mediated Activation of Immune Responses in Humans

Extensive analyses were performed to evaluate mechanisms-of-action of the oncolytic and immunotherapeutic vaccinia virus Pexa-Vec in patients. These include oncolysis (15, 77, 78), acute vascular disruption (29) as well as anti-tumor immune response induction. Pexa-Vec was engineered to express GM-CSF to stimulate white blood cell production and activate DCs. Detectable concentrations of GM-CSF in plasma were measured 4–15 days after treatment and associated with increased neutrophil, monocyte, and eosinophil production in patients receiving iv or it iPexa-Vec (77, 78). Inflammatory cell recruitment to tumors was confirmed on biopsy following Pexa-Vec administration in patients with melanoma (79, 80). Furthermore, functional anti-cancer immunity of Pexa-Vec treatment was demonstrated in patients by measuring induction of antibody-mediated complement-dependent cytotoxicity (CDC) utilizing a panel of tumor cell lines of different histologies (30). Low concentrations of serum *ex vivo* incubated with tumor cells resulted in a dramatic reduction in tumor cell viability; when normal cells did not exhibit decreased viability. This activity was shown to be dependent on both active complement as well as IgG antibody. Reproducible CDC activity was also observed in a Phase II study in HCC patient (18). Furthermore, T-cell responses to β-galactosidase peptides were detected in HCC patients treated with Pexa-Vec, as shown by ELISPOT analysis. In that way, the proof-of-concept provides that T-cell responses can be induced to transgenes encoded by oncolytic vaccinia viruses (18).

Talimogene laherparepvec is an oncolytic immunotherapy comprising a modified HSV type 1 engineered to selectively replicate in tumor cells and to express the immune-stimulating cytokine GM-CSF, while retaining sensitivity to antiherpetic agents (16). Local effects after intralesional injection include selective lysis of tumor cells and subsequent release of tumor antigen, as well as secretion of GM-CSF into the local environment, which results in the stimulation and maturation of DCs (32, 81). Antigen presentation by stimulated DCs to CD4^+^ and CD8^+^ cells may induce an adaptive systemic immune response (16, 82, 83). Recently a randomized, Phase III trial of talimogene laherparepvec in patients (pts) with unresected melanoma with regional or distant metastases (OPTiM) met its primary endpoint, demonstrating a significant improvement in durable response rate (defined as partial or complete responses that were maintained for ≥6 months starting within 12 months) versus GM-CSF alone (16 versus 2%, *p* < 0.0001) (17). Overall response rate was also higher in the talimogene laherparepvec arm (26.4 versus 5.7%, *p* < 0.0001). Subjects treated with talimogene laherparepvec showed a tolerable safety profile with the only grade 3/4 adverse event that occurred in >2% of patients being cellulitis (2.1%). A trend toward improved overall survival was seen based on a planned interim analysis (17). The primary overall survival results are pending. Evidence of durable responses together with the safety profile of talimogene laherparepvec supports evaluation of combinations with other immunotherapies, such as high-dose IL-2 or immune checkpoint blockade and with radiation therapy, chemotherapy, and/or targeted therapies that might amplify the anti-tumor response generated by talimogene laherparepvec (32).

## Discussion: Potential of the Immunovirotherapy Concept

Despite recent improvements in surgical, locoregional, and systemic therapies, the prognosis of patients with gastrointestinal, hepatobiliary, and pancreatic cancers remains dismal, and treatment is limited to palliation in the majority of patients. These limitations indicate an urgent need for novel therapeutic strategies (13, 64, 66, 84). Combinations of oncolytic viruses with new targeted therapies draw much attention. It is however necessary to proceed with caution, as these therapies may interfere with pathways, which are needed for replication of genetically modified viruses. It was demonstrated that by interacting with the EGFR/RAS/RAF pathway, sorafenib inhibits replication of Pexa-Vec in liver cancer, when applied in combination. This is not surprising as Pexa-Vec replication is in part dependent on the EGFR/RAS/RAF pathway (85). Nevertheless, sequential therapy with Pexa-Vec followed by sorafenib resulted in decreased tumor perfusion and was associated with objective tumor responses for HCC (85). It is noteworthy that some oncolytic viruses such as parvovirus H-1PV also have potential to inhibit neo-angiogenesis. Therefore, OV-based combination treatments targeting both tumor cell proliferation and tumor angiogenesis represent a promising strategy for impeding the growth of various cancers (25).

Besides their low expression of TAA and low immunogenicity, tumors can induce an immune tolerance milieu by releasing anti-inflammatory cytokines such as IL-10 or TGF-β or recruiting Tregs to their microenvironment (86). T-cell activation relies on both, recognition of major histocompatibility complex (MHC) molecules by the T-cell receptor (TCR), and on costimulatory signals. Depending on the type of costimulatory receptor, T-cells can be activated or become anergic. For example, T-cell activation was prevented by engagement of CTLA-4 receptors with CD80 or CD86. In contrast, engagement of CD80 or CD86 with CD28 induced T-cell activation, often with a low affinity (87). Thus, a promising therapeutic option to achieve strong anti-tumor immune responses is the use of monoclonal antibodies against CTLA-4 and PD-1 alone or in combination. Herein, the constitutive expression of CTLA-4 and PD-1 on Tregs may play a crucial role in inhibiting anti-tumor T-cell responses. Tregs are often found in the peripheral blood of cancer patients and in the tumor microenvironment. These cells suppress an optimal anti-tumor immune response by preventing infiltrating CD8^+^ T-cells from proliferating and producing cytolytic granules (88). BMS developed an anti-CTLA-4 monoclonal antibody named ipilimumab and an anti-PD-1 monoclonal antibody named nivolumab. Both antibodies were already tested in Phase III trials and found to achieve clinically significant benefits in median overall survival (89, 90). First pre-clinical studies of the combination of these antibodies to achieve blockade of both CTLA-4 and PD-1 showed increased tumor infiltration by CD4^+^ and CD8^+^ T-cells, enhanced IFNγ and TNFα production, and reduced amounts of Tregs (91). A Phase I study of nivolumab and ipilimumab combination in advanced melanoma patients showed an outstanding activity in 65% of patients with an objective response rate of 40% (92). As part of their further development and mechanistic understanding, these antibodies against immune check points would certainly deserve to be combined with OV in order to optimize anti-tumor immune responses. Preliminary data from a Phase Ib trial combining talimogene laherparepvec with ipilimumab indicated that the combination was tolerable and devoid of unexpected toxicities (93). Exploiting these combinations represents a promising strategy to bring oncolytic viruses from bench to bedside and to establish oncolytic virotherapy as a new effective immunotherapeutic approach.

## Conflict of Interest Statement

The authors declare that the research was conducted in the absence of any commercial or financial relationships that could be construed as a potential conflict of interest.

## Key Concepts

- **Key concept_1_**: There is a consistent need for immunotherapies in the treatment of human cancer.
- **Key concept_2_**: Oncolytic viruses reduce tumor burden and show first clinical results in humans.
- **Key concept_3_**: Oncolytic viruses, such as parvovirus H-1PV, induce effective anti-tumor immune responses.
- **Key concept_4_**: Combinations of oncolytic viruses with immunotherapeutics are likely to achieve enhanced immune activation.
